# Implementation and Access Gaps in Infectious Disease Screening for Migrants in Europe: A Structured Review with Thematic Synthesis From the ESGITM Group

**DOI:** 10.5334/aogh.5363

**Published:** 2026-09-02

**Authors:** Rosa Buonamassa, Nikolaos Markou-Pappas, Alberto Matteelli, Ferenc Balázs Farkas, Muhammad Asaduzzaman, Luisa Frallonardo, Botond Lakatos, Annalisa Saracino, Alba Cuxart-Graell, Nathan Bertelsen, Christian Wejse, Alexandre Raphael Meduri, Giacomo Guido, Irene Losada, Pietro Locantore, Roberta Iatta, Caroline Rönnberg, Francesco Di Gennaro

**Affiliations:** 1 Department of Internal Medicine and Medical Therapy, University of Pavia (UniPV), Pavia, Italy; 2 General Surgery Residency Program, University of Pavia, Pavia, Italy; 3 Institute of Infectious and Tropical Diseases, Department of Clinical and Experimental Sciences, WHO Collaborating Centre for TB Prevention, University of Brescia, Brescia, Italy; 4 Pediatric Center, Semmelweis University, Budapest, Hungary, Insititute of Medical Microbiology, Semmelweis University, Budapest, Hungary; 5 Department of Community Medicine and Global Health, Institute of Health and Society, Faculty of Medicine, University of Oslo, Oslo, Norway; 6 Clinic of Infectious Diseases, Department of Precision and Regenerative Medicine and Ionian Area, University of Bari Aldo Moro, Italy; 7 Department of Hematology and Infectious Diseases, School of Infectious Diseases, Semmelweis University, Budapest, Hungary; 8 Barcelona Institute for Global Health (ISGlobal), Hospital Clínic, University of Barcelona, Barcelona, Spain; 9 Minnesota Asylum Collaborative, Global Medicine, University of Minnesota Medical School, USA; 10 Department of Public Health, Faculty of Health Science, Aarhus University, Aarhus, Denmark; 11 Section of Dermatology and Venereology, Department of Precision and Regenerative Medicine and Ionian Area (DiMePre-J), University of Bari Aldo Moro, Bari, Italy; 12 Unit of Endocrinology, Department of Translational Medicine and Surgery, Fondazione Policlinico “A. Gemelli” IRCCS, Università Cattolica del Sacro Cuore, Rome, Italy; 13 Interdisciplinary Department of Medicine, University of Bari, Bari, Italy; 14 Department of Microbiology, Unit for Parasitology, Public Health Agency of Sweden, Solna, Sweden

**Keywords:** migrants, refugees, infectious disease screening, Europe, access to care, continuity of care, implementation, health equity

## Abstract

*Background:* Infectious disease screening is widely recommended for migrants in Europe, yet implementation remains inconsistent across countries and settings. While guidance has focused on which infections to screen for, less attention has been paid to how screening is delivered, accessed, and linked to care. This structured review with thematic synthesis aimed to synthesize evidence on implementation gaps, barriers, and facilitators, with particular attention to differences across the migratory cycle.

*Methods:* A structured search was conducted across PubMed, Scopus, Web of Science, and Google Scholar for publications from January 2000 to February 2026, addressing infectious disease screening or related migrant health assessments in European settings. Of 49 included publications, 13 empirical studies formed the primary analytical basis and were quality-appraised using the Mixed Methods Appraisal Tool (MMAT, 2018); the remaining 36 contextualizing publications were used to interpret findings. Eligibility screening was performed independently by two reviewers.

*Results:* Six interconnected thematic domains shaped screening implementation: administrative and legal barriers; communication, language, and cultural mediation; trust, stigma, and perceived coercion; organizational capacity and fragmented care pathways; the role of NGOs and community actors; and contextual differences across the migratory cycle. Barriers varied across the screening-to-care cascade: communication barriers and mistrust primarily affected uptake; legal and organizational barriers constrained linkage to diagnosis and treatment; and legal instability and fragmented services undermined continuity of care. First-arrival settings were characterized by systematic screening and high service pressure; long-term settlement contexts relied more heavily on primary care capacity, trust, and legal entitlement.

*Conclusions:* Implementation gaps are driven less by the absence of clinical guidance than by structural, organizational, and relational barriers limiting access, linkage, and continuity. Screening should be embedded within integrated, migrant-centered care pathways supported by cultural mediation, trusted community actors, and separation from immigration enforcement.

## Introduction

Infectious disease screening has become a central component of public health strategies, commonly justified by the dual aim of ensuring early diagnosis and treatment for individuals at increased epidemiological risk and contributing to health equity across European populations. Among these populations, migrants represent a group with specific vulnerabilities. Evidence suggests that migrants may experience a higher mortality risk from infectious diseases with a Standardized Mortality Ratio (SMR) of 2.4, may present later to health services, and may have poorer clinical outcomes, factors that can contribute to onward transmission within migrant communities [1–4].

In response to these challenges, a substantial body of international and regional guidance has been developed. Organizations such as the World Health Organization (WHO) and the European Centre for Disease Prevention and Control (ECDC) have issued recommendations to guide infectious disease screening and vaccination practices among migrants, asylum seekers, and refugees [5–8]. These policy efforts are aligned with the 2030 Sustainable Development Goals, particularly the commitment to “leave no one behind,” which calls on United Nations member states to ensure universal health coverage so that all populations can access essential health services [6, 9, 10].

Despite the availability of such guidance, substantial variation persists in how infectious disease screening is implemented across European countries and healthcare settings [11, 12]. Emerging evidence indicates that the effectiveness of screening programs is often constrained not by diagnostic tools or clinical knowledge, but by challenges related to implementation, access to care, and continuity along the screening–treatment pathway [6]. European countries differ markedly in the extent and type of healthcare to which migrants are legally entitled, particularly migrants with irregular status, resulting in heterogeneous access to preventive services [12]. Structural and organizational barriers, including legal restrictions, administrative complexity, language barriers, and mistrust of institutions, frequently limit migrants’ ability to access screening and subsequent care [13, 14].

Importantly, these challenges are not uniform across the migratory trajectory. First-arrival settings, such as reception centers or temporary accommodation facilities, are often characterized by high service pressure, systematic or mandatory screening practices, and limited continuity of care [3]. In contrast, longer-term settlement contexts rely more heavily on primary care and community-based services, where access to screening is frequently opportunistic and dependent on health system navigation, trust, and legal entitlement [3, 15]. Understanding how screening policies operate across these different stages of the migratory cycle is therefore essential to identifying persistent implementation gaps [16].

These contextual differences across the migratory cycle have increasingly informed policy debates, highlighting the limitations of disease-specific and setting-bound screening approaches. Recent guidance has increasingly emphasized the need to move beyond disease-specific, siloed approaches toward more holistic and integrated models of care. In particular, ECDC guidance recommends that clinicians and public health programs adopt integrated, multi-disease screening and vaccination strategies for newly arrived migrants [6, 7, 16]. Building on this perspective, an integrated roadmap for the prevention and treatment of infectious diseases among migrants in Europe has been proposed, highlighting the importance of co-designing programs with migrant communities, embedding screening within health systems, removing barriers to access, ensuring high acceptability, and strengthening linkage to care across regions [7, 8, 14, 17–19].

While previous reviews have examined the epidemiology of infectious diseases among migrants and the clinical effectiveness of screening, fewer studies have systematically explored how screening policies are translated into practice and experienced by migrants and healthcare providers [19]. This review, therefore, aims to synthesize existing evidence on the implementation of infectious disease screening for migrants in Europe, with a specific focus on access to care, contextual barriers and facilitators, and differences across stages of the migratory cycle.

## Methods

This review was designed as a structured review with thematic synthesis of the published literature. The term “structured” is used here to reflect a review that follows a pre-specified search strategy, explicit eligibility criteria, and independent dual screening, while acknowledging that it was not prospectively registered and incorporated heterogeneous publication types beyond those permissible in a fully systematic review. Thematic synthesis was selected because the heterogeneous study designs, populations, and outcome measures encountered in the eligible literature precluded statistical pooling, and because the primary objective was to construct an explanatory framework of implementation barriers and facilitators rather than to estimate prevalence or effectiveness [20], which maps the breadth of available evidence without developing analytical themes from primary data. This approach was preferred over a scoping review, which maps the breadth of available evidence without developing analytical themes from primary data, and over meta-ethnography or qualitative evidence synthesis, which require a more homogeneous qualitative evidence base than was available here, given that our synthesis incorporates observational, mixed-methods, and policy literature alongside qualitative studies. A rapid review was considered inappropriate given the complexity and Europe-wide scope of the research questions.

The review addressed the following questions:

1. How are infectious disease screening policies for migrants implemented in practice across European healthcare settings?
2. What barriers and facilitators influence access to screening and subsequent care?
3. How do implementation challenges differ between first-arrival and long-term settlement contexts?

### Search strategy

A structured literature search was conducted in PubMed, Scopus, Web of Science, and Google Scholar. The search covered the period from January 1, 2000 to February 1, 2026. The year 2000 was chosen as the lower time limit because it predates key transformations in the European migration landscape—including EU enlargement, the development of contemporary migration and asylum frameworks, and the establishment of modern infectious disease screening guidance—after which the evidence base becomes both more relevant and more comparable across European settings. Search strategies combined controlled vocabulary, where applicable, and free-text terms related to four main domains:

- migrants and people on the move;
- infectious disease screening and related health assessments;
- implementation, access, barriers, facilitators, and continuity of care; and
- European settings.

A sample search strategy for PubMed was as follows: (“migrant” OR “refugee” OR “asylum seeker*” OR “undocumented migrant” OR “newly arrived migrant”) AND (“infectious disease screening” OR screening OR vaccination OR “health assessment” OR “health examination”) AND (implementation OR access OR uptake OR barrier OR facilitator OR acceptability OR continuity OR “linkage to care” OR “care pathway”) AND (Europe OR European OR “EU” OR “EU/EEA”).

### Eligibility criteria

Studies were eligible for inclusion if they:

- focused on migrants, refugees, asylum seekers, or migrants with irregular status;
- addressed infectious disease screening, vaccination-related screening activities, or related migrant health assessments with implications for screening access and follow-up;
- examined implementation processes, access to care, contextual barriers or facilitators, acceptability, or continuity of care;
- were conducted in European countries or were directly relevant to European healthcare systems;
- used qualitative, mixed-methods, observational, implementation-oriented, review, policy, or conceptual designs capable of informing implementation-related questions;
- were published in English.

Two key terms used throughout this review warrant explicit definition. Acceptability was understood broadly to encompass the perspectives of migrants, asylum seekers, and refugees regarding screening processes and services, as well as those of healthcare professionals and other key stakeholders involved in screening delivery. The term “newly arrived migrants” was not defined by a uniform temporal threshold across included studies, as definitions varied considerably; for the purposes of this review, “newly arrived” is used descriptively to refer to migrants in the early phases of their migratory trajectory, typically accessed through first-arrival or reception settings, as distinct from those in longer-term settlement contexts.

Studies were excluded if they:

- focused exclusively on clinical effectiveness, diagnostic performance, or epidemiological prevalence without discussion of implementation or access;
- were conducted in non-European settings without clear applicability to Europe;
- were not available in English;
- were editorials, conference abstracts, or commentaries without sufficient empirical or conceptual detail.

To ensure methodological rigor, title and abstract screening and full-text eligibility assessment were each performed independently by two reviewers [RB, NMP]. Any discrepancies arising at either stage were resolved through discussion and mutual consensus. No formal inter-rater reliability coefficient was calculated, in keeping with accepted practice for thematic synthesis reviews.

For the purposes of thematic synthesis, included studies were further classified into two categories. Studies were classified as empirical and implementation-informative if they: (i) collected primary data directly from participants, clinical records, or healthcare settings; (ii) focused on real-world implementation, access, or experience of screening in a specific identifiable context; and (iii) employed a qualitative, mixed-methods, or observational design. Studies not meeting these criteria—including systematic reviews, scoping reviews, policy documents, guidelines, commentaries, and conceptual papers—were classified as contextualizing evidence and used to interpret and situate the findings of the thematic synthesis rather than as primary analytical inputs.

Data were charted iteratively, extracting information on study characteristics, screening context, reported barriers and facilitators, stakeholder perspectives, and relevance to stages of the migratory cycle. A binary coding matrix was developed for the 13 empirical studies to record the presence or absence of key themes identified during the thematic synthesis. Given the heterogeneity of the evidence base, findings were synthesized using a narrative and thematic approach.

### Quality appraisal

The methodological quality of the 13 empirical studies was appraised using the Mixed Methods Appraisal Tool (MMAT, 2018 version), which accommodates qualitative, quantitative descriptive and mixed methods designs through a common set of screening criteria and five design-specific criteria scored as Yes, No, or Cannot Tell. No studies were excluded on the basis of quality; scores were used to inform the interpretation of confidence in individual findings. Results of the quality appraisal are presented in Supplementary Table S1.

### Data extraction

Data were charted iteratively using a structured extraction form developed by the authors. Extracted information included: author and year; country; study setting; target population; screening focus; study design; migratory context; and key findings related to implementation, access, barriers, facilitators, acceptability, trust, communication, organizational capacity, and continuity of care.

### Data synthesis

Given the heterogeneity of study designs and outcomes, findings were synthesized using a narrative thematic approach. The authors reviewed the included literature and identified recurring implementation-related patterns across studies. Although themes were developed inductively through iterative reading of the included literature, the analytic process was informed by established frameworks of healthcare access, in particular the patient-centered access model proposed by Levesque et al. [21]. The resulting thematic domains map broadly onto the five dimensions of service accessibility identified in that framework—approachability, acceptability, availability and accommodation, affordability, and appropriateness. Trust, stigma, and perceived coercion, and the specific role of non-governmental organizations (NGOs) and community actors, emerged as contextually relevant additions reflecting the particular vulnerabilities of migrant populations in European healthcare systems. This approach is consistent with a hybrid inductive-deductive thematic synthesis, in which themes emerge primarily from the data while being interpreted within an existing theoretical framework [20].

A binary coding matrix was then developed for the subset of empirically informative studies to record the presence or absence of key themes across articles. The final thematic framework included the following domains:

- administrative and legal barriers;
- communication, language, and cultural mediation;
- trust, stigma, and perceived coercion;
- organizational capacity and fragmented care pathways;
- the role of NGOs and community actors;
- contextual differences across the migratory cycle.

Impact ratings assigned to each thematic domain were determined by authorship consensus, informed by the frequency and consistency with which each barrier or facilitator was reported across the 13 empirical studies as recorded in the binary coding matrix.

## Results

**Figure 1:**
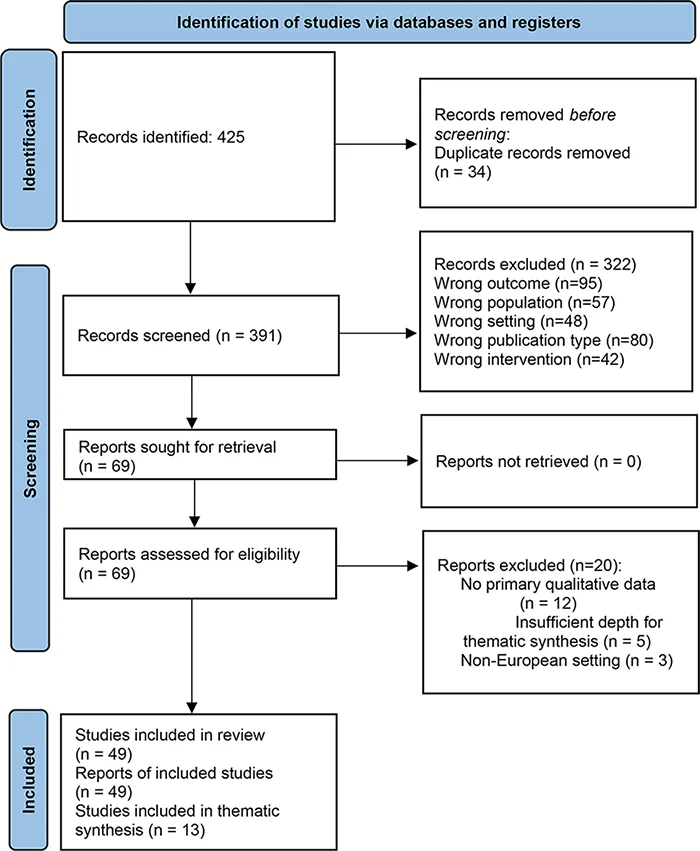
PRISMA flow diagram of study identification, screening, eligibility assessment, and inclusion.

The search identified 425 records; following deduplication and screening, 69 reports were assessed for full-text eligibility, of which 20 were excluded because they lacked primary data, provided insufficient depth for thematic synthesis, or were conducted in non-European settings (Figure 1).

A total of 49 publications met inclusion criteria. Of these, 13 empirical studies—defined as publications collecting primary data in real-world implementation contexts using qualitative, mixed-methods, or observational designs—formed the primary analytical basis of the thematic synthesis (Table 1). The remaining 36 contextualizing publications—comprising systematic reviews (n = 10), qualitative and observational studies (n = 7), policy documents and guidelines (n = 9), and conceptual or methodological papers (n = 10)—were used to situate and interpret findings and are described in full in Supplementary Table S2.

**Table 1:** Characteristics of included empirical studies informing the thematic synthesis.

AUTHOR	COUNTRY	SETTING	POPULATION	SCREENING FOCUS	STUDY DESIGN	MIGRATORY CONTEXT
Nkulu-Kalengayi et al. [22]	Sweden	Reception and primary care services	Newly arrived migrants	Health screening (general)	Qualitative interviews	First arrival
Louka et al. [23]	Greece, Netherlands	Reception centers	Asylum seekers	Vaccination and infectious disease screening	Qualitative interviews	First arrival
Kang et al. [24]	United Kingdom	Primary care	Asylum seekers and refugees	Access to primary healthcare	Qualitative community-based study	Long-term settlement
Khanom et al. [25]	United Kingdom (Wales)	Primary care and community services	Asylum seekers and refugees	Access to healthcare services	Qualitative interviews (focus groups)	Long-term settlement
Delilovic et al. [26]	Sweden	Health examination services (HE)	Asylum seekers	Health examinations including screening	Qualitative interviews (providers)	First arrivals
Gonçalves et al. [27]	Spain	Primary care	Migrant patients	Multi-disease screening	Qualitative study (GPs)	Long-term settlement
Seedat et al. [14]	United Kingdom	Community and primary care	Migrant communities	Infectious disease screening	Qualitative interviews	Long-term settlement
Scott et al. [28]	United Kingdom	Primary care	Refugees and asylum seekers	Primary healthcare access	Qualitative study	Long-term settlement
Carter et al. [29]	United Kingdom	Primary care	At-risk migrant patients	Multi-disease screening and catch-up vaccination	Mixed-methods	Long-term settlement
Moffat et al. [30]	United Kingdom	Community health services	Refugees and asylum seekers	Health access facilitation	Qualitative evaluation	Long-term settlement
Marrone et al. [31]	Italy	Reception centers and regional services	Refugees and asylum seekers	NTDs and infectious disease screening	Observational, prospective prevalence study	First arrival
Kortas et al. [32]	Germany	Reception center	Newly arrived asylum seekers	Infectious disease screening	Retrospective analysis	First arrival
Boye et al. [33]	Denmark	Community health services	Migrants	HIV testing	Qualitative study	Long-term settlement

Quality appraisal of all included empirical studies was conducted using the MMAT is reported in full in Supplementary Table S1.

Migratory context reflects the setting and population addressed by each study. “First arrival” refers to studies conducted in reception or entry-point settings; “Long-term settlement” refers to studies conducted in primary care or community settings among migrants with established residence. Temporal thresholds for “newly arrived” varied across studies.

These studies included qualitative, mixed-methods, and observational designs and were interpreted in light of the broader evidence base included in the review. Geographically, the evidence base was concentrated in the United Kingdom (n = 6), followed by Sweden (n = 2), with single studies from Italy, Spain, Germany, Denmark and a comparative study spanning Greece and the Netherlands. Studies were conducted across a range of healthcare settings, including primary care and community services (n = 7), first-arrival and reception center contexts (n = 5), and one study addressing both settings. A more detailed breakdown of studies by country and setting is provided in Table 1.

In terms of population and scope, the majority of studies focused on migrants, asylum seekers, or refugees, with several explicitly addressing populations with irregular status. Screening interventions included both single-disease approaches (e.g., tuberculosis, hepatitis B or C, HIV) and multi-disease health assessments. Studies addressed different stages of the migratory trajectory, allowing comparison between first-arrival settings and longer-term settlement contexts.

### Thematic synthesis of findings

In the following thematic synthesis, the 13 empirical studies listed in Table 1, denoted throughout by an asterisk (*), form the primary analytical basis; the distribution of themes across these studies is presented in Table 2, while the broader contextualizing literature is used to situate and interpret findings.

**Table 2:** Binary coding matrix of barriers, facilitators, and migratory-context themes across empirical studies informing the thematic synthesis.

AUTHOR	A	C	T	O	N	F	L
Nkulu-Kalengayi et al. [22]	Y	Y	Y	N	N	Y	N
Louka et al. [23]	N	Y	Y	N	N	Y	N
Kang et al. [24]	Y	Y	Y	Y	N	N	Y
Khanom et al. [25]	Y	Y	Y	N	N	N	Y
Delilovic et al. [26]	Y	N	N	Y	N	Y	N
Gonçalves et al. [27]	N	Y	N	Y	N	N	Y
Seedat et al. [14]	N	Y	Y	N	Y	N	Y
Scott et al. [28]	Y	N	Y	Y	N	N	Y
Carter et al. [29]	Y	N	N	Y	Y	N	Y
Moffat et al. [30]	Y	N	Y	N	Y	N	Y
Marrone et al. [31]	Y	N	N	Y	N	Y	N
Kortas et al. [32]	Y	N	N	Y	N	Y	N
Boye et al. [33]	Y	N	N	N	N	N	Y
**Total number (n/13)^a^**	**10**	**6**	**7**	**7**	**3**	**5**	**8**

Coding matrix of empirical studies (Y = yes, N = no). Themes: A = Administrative / legal barriers, C = Communication, language, cultural mediation, T = Trust, stigma, perceived coercion, O = Organizational capacity / fragmented pathways, N = Role of NGOs / community actors, F = First-arrival context explicitly addressed, L = Long-term settlement context explicitly addressed.

#### Theme 1: Administrative and legal barriers to screening and care

Administrative and legal barriers emerged, across the breadth of included literature, as central determinants of access to infectious disease screening and subsequent care [4, 18]. Multiple studies reported that entitlement restrictions, documentation requirements, and uncertainty regarding legal status limited migrants’ ability to engage with screening programs, particularly beyond the initial point of contact with health services [18, 22*, 24*, 25*, 34]. These barriers were described as especially consequential for migrants with irregular status and those transitioning between care settings [4, 14*, 30*, 34].

Evidence highlighted that such constraints did not only affect initial access to screening, but also undermined linkage to treatment and continuity of care [26*, 29*, 35]. Even where screening was formally available, fragmented administrative processes and unclear responsibilities frequently resulted in loss to follow-up [19, 31*, 32*]. These dynamics were observed across different European contexts and were reinforced in settings experiencing high migratory pressure, where services operated under constrained conditions [18, 26*, 27*, 36].

#### Theme 2: Communication, language, and cultural mediation

Communication difficulties, language differences, and limited cultural mediation were consistently identified as key barriers influencing screening uptake and acceptability [14*, 22*, 23*, 35]. Studies reported that limited access to professional interpreters and culturally appropriate information hindered migrants’ understanding of screening procedures, purposes, and implications [13, 16, 19, 23*, 30*, 36].

From the perspective of healthcare providers, inadequate communication was associated with increased time pressure and uncertainty in clinical encounters [26*, 27*, 34, 36]. From migrants’ perspectives, lack of clear information contributed to confusion, anxiety, and reluctance to engage with screening [4, 22*, 23*]. These challenges were found to affect not only first-arrival encounters, but also longer-term interactions within primary care and community services [27*, 28*, 35].

#### Theme 3: Trust, stigma, and perceived coercion

The degree of trust migrants held toward healthcare institutions emerged as a critical factor shaping their engagement with screening programs [4, 14*, 22*, 35]. Several studies highlighted that screening was sometimes perceived as coercive or linked to migration control, particularly in first-arrival contexts where participation was mandatory or poorly explained [19, 22*, 23*]. Such perceptions contributed to fear, stigma, and mistrust, undermining acceptability and engagement [14*, 20, 25*, 36, 37].

The literature further indicated that historical experiences of discrimination or marginalization influenced migrants’ willingness to disclose information and participate in preventive interventions [4, 24*, 25*, 30*, 34]. Trust-related issues were closely intertwined with communication barriers and legal insecurity, reinforcing disengagement from screening and follow-up care [22*, 26*, 35]. These patterns were consistently reported across different national contexts [6, 14*, 16, 23*, 28*].

#### Theme 4: Organizational capacity and fragmented care pathways

Recurrent deficits in organizational capacity and pervasive fragmentation of care pathways were widely reported as obstacles to effective screening implementation [4, 19, 26*, 27*, 35]. Studies described insufficient staffing, lack of training, and unclear operational guidance as recurrent challenges for healthcare providers [26*, 27*, 34, 36]. Screening programs were often implemented as stand-alone activities, with limited integration into broader healthcare pathways [16, 18, 19, 27*].

Fragmentation was particularly evident at the interface between screening and treatment, where referral mechanisms and follow-up responsibilities were frequently unclear [19, 29*, 30*, 34, 35, 38]. In high-pressure contexts such as reception centers, emergency-driven service organization prioritized immediate needs over continuity of care, while in longer-term settlement settings, migrants faced difficulties navigating complex health systems [19, 24*, 25*, 26*, 32*, 35]. These challenges contributed to attrition along the screening–care cascade [4, 27*, 31*].

#### Theme 5: Role of NGOs and community actors

A bridging role for NGOs and community actors was repeatedly identified across the evidence base, positioning them as key facilitators of access to screening and care [14*, 18,19, 25*, 30*]. Across diverse settings, NGOs played a bridging role by providing outreach, information, interpretation, and navigation support, particularly for migrants excluded from formal healthcare entitlements [4, 14*, 25*, 28*, 34].

However, the literature also noted that the involvement of NGOs was often informal and dependent on short-term funding or local initiatives [19, 27, 30*]. This reliance raised concerns regarding sustainability, equity, and consistency of service provision [14*, 18, 27*]. Several studies emphasized the need for stronger integration of community actors within formal screening strategies and health system planning [4, 6, 14*, 17, 27, 36].

Beyond the empirical literature, policy reviews and implementation frameworks consistently identify NGOs and community organizations as essential intermediaries between health systems and migrant communities, particularly in contexts where formal services are inaccessible or mistrusted [6, 7, 19]. Despite this recognized importance, the integration of NGOs within formal screening strategies remains largely informal and funding-dependent, with sustainability representing a persistent challenge across European contexts [18, 34].

#### Theme 6: Contextual differences across the migratory cycle

The stage of the migratory cycle shaped implementation challenges considerably, with distinct patterns observed in first-arrival and long-term settlement contexts [4, 19, 22*, 23*, 26*]. First-arrival settings were characterized by systematic or mandatory screening, high service pressure, and limited continuity of care [19, 26*, 31*]. In contrast, longer-term settlement contexts relied more heavily on opportunistic screening within primary care, where access was shaped by trust, navigation capacity, and legal entitlement [24*, 27*, 28*, 35].

**Figure 2:**
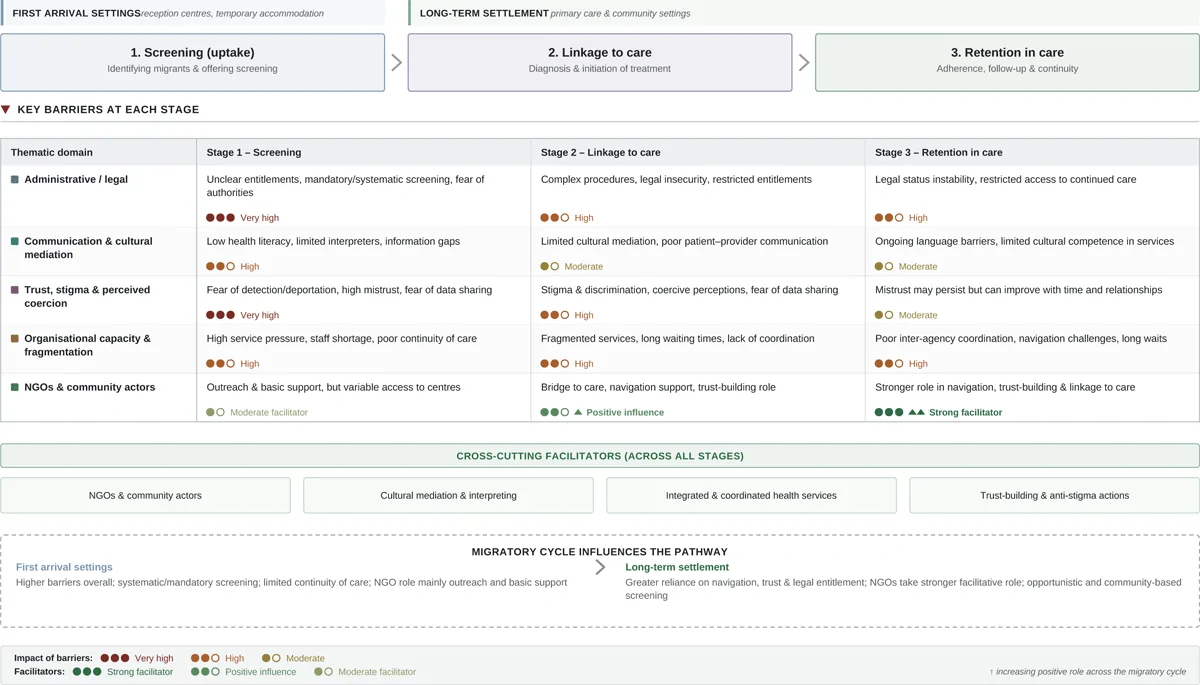
Representative cascade model of barriers and facilitators influencing infectious disease screening, linkage to care, and retention in care among migrants across the migratory cycle. Barriers organized by thematic domain across three sequential care stages; impact severity and facilitating influence indicated by colored dots.

Despite these contextual differences, barriers related to access and continuity of care persisted across settings [14*, 18, 25*, 39]. The literature suggested that vulnerabilities accumulated over time, particularly for migrants experiencing prolonged legal precarity or repeated transitions between services [4, 22*, 30*, 34] (Figure 2). These findings underscore the importance of considering screening implementation as a dynamic process unfolding across the migratory trajectory rather than as a single intervention [4, 23*, 27, 35].

## Discussion

This review synthesized evidence on the implementation of infectious disease screening for migrants in Europe, identifying six interconnected thematic domains that shape screening access and effectiveness. The thematic domains identified in this review align closely with the patient-centered access framework of Levesque et al. [21], with trust and the role of community actors emerging as contextually specific additions that reflect structural vulnerabilities not fully captured by existing models. The findings consistently indicate that implementation gaps arise not from insufficient clinical guidance, but from structural and contextual factors embedded within health systems and migration governance [4, 6]. Administrative barriers, communication challenges, trust dynamics, organizational fragmentation, and the informal role of community actors emerged as mutually reinforcing dimensions of a complex implementation landscape.

A key contribution of this review lies in examining screening implementation across the migratory cycle, rather than within isolated settings or disease-specific programs. While previous systematic reviews have assessed the clinical effectiveness of screening interventions [14] or synthesized evidence on accessibility and acceptability [20], fewer studies have explicitly traced how barriers evolve as migrants transition from first-arrival contexts to longer-term settlement. The findings suggest that vulnerabilities accumulate over time, particularly for those experiencing prolonged legal precarity or repeated service transitions [15, 39].

The interconnection among barriers warrants particular attention. Legal restrictions and documentation requirements not only limit initial access but also generate uncertainty that undermines trust and engagement with health services [11, 12]. Communication difficulties compound these dynamics, transforming screening encounters into experiences perceived as opaque or coercive, particularly when interpretation services are unavailable or cultural mediation is lacking [35]. These patterns align with the 3C model proposed by Brandenberger et al., which identifies communication, continuity, and confidence as core challenges in healthcare delivery to migrants [35]. Organizational fragmentation further amplifies these barriers, as unclear referral pathways and weak integration between screening and treatment services result in loss to follow-up even when initial access is achieved [19, 34].

These findings carry direct implications for policy and practice. Efforts to strengthen screening should explicitly separate healthcare provision from immigration enforcement, as the conflation of these domains undermines trust and discourages engagement with preventive services [4]. Formal integration of NGOs within screening strategies is needed, moving beyond informal arrangements toward sustainable partnerships embedded in health system planning [7, 18]. Screening should be reconceptualized as a longitudinal process within broader care pathways, with attention to continuity across the migratory trajectory rather than isolated diagnostic events [7,15]. Policy frameworks should address structural barriers alongside clinical protocols, ensuring that entitlement rules and administrative procedures do not undermine the public health objectives that screening programs are designed to achieve. These implementation gaps have direct relevance to the 2030 Sustainable Development Agenda. The SMR of 2.4 for infectious diseases among migrants—substantially higher than in host populations—suggests that the “leave no one behind” commitment is not being met for this group [1, 9]. Addressing the structural barriers identified in this review is therefore not only a matter of clinical and public health priority, but a precondition for fulfilling internationally binding commitments to health equity [6].

Viewed through an equity lens, the findings underscore a fundamental tension inherent in migrant health screening: programs nominally designed to protect and benefit migrants are often experienced as surveillance mechanisms or administrative checkpoints rather than as expressions of care. This dynamic is documented across multiple European contexts. In Sweden, asylum seekers are offered health examinations including infectious disease screening upon arrival, yet their entitlement to subsequent care is legally restricted to “care that cannot be postponed”—meaning that individuals identified with treatable chronic infections may receive a diagnosis without access to treatment [40]. Similarly, in the United Kingdom, the progressive entanglement of NHS services with immigration enforcement under successive hostile environment policies has been shown to deter migrants with irregular status from engaging with preventive care, including screening, even when legally entitled to it [41, 42]. In both contexts, screening functions less as an entry point to care than as an administrative checkpoint—a dynamic that transforms it into a largely symbolic or even counterproductive exercise. The ethical implications of this gap are substantial. Screening a population for a treatable condition without ensuring access to treatment not only fails to benefit the individual but may also generate harm through stigmatization, anxiety, or breaches of confidentiality in politically hostile environments. A genuinely rights-based approach to screening requires that it be embedded within a continuum of care that is accessible, voluntary, and free from any link to migration enforcement [43]. Addressing this structural contradiction is therefore not merely a question of operational efficiency, but of medical ethics and human rights.

From a health systems perspective, the evidence points to a structural mismatch between the design of screening programs and the complex, non-linear trajectories through which migrants move within and across health systems. Most existing frameworks are built around single encounters—typically at first arrival—and are not designed to account for the longitudinal nature of health risk or the discontinuities that characterize migration [15]. Migrants may undergo initial screening in a reception center, be referred for follow-up in a different region, and subsequently register with primary care under a provider with no access to prior records—creating structural gaps that individual clinical encounters cannot bridge [39]. Integrated multi-disease screening models, such as those piloted in Spain and the United Kingdom [27], offer a promising direction, though their scalability beyond research settings remains uncertain. Health information systems capable of supporting continuity across settings and time, alongside training and resourcing of primary care providers, are necessary—if often overlooked—components of effective screening infrastructure [43]. Strengthening the capacity of health systems at all levels to respond to the specific and evolving needs of migrant populations is thus a precondition for translating policy commitments into meaningful health gains.

This review has limitations. The heterogeneity of included studies, spanning different countries, populations, and settings, limits the generalizability of specific findings. The empirical evidence base is also geographically concentrated, with most studies originating from the United Kingdom and Sweden. Notably, the four EU countries hosting the largest foreign-born populations—Germany, France, Spain, and Italy, collectively representing 66.7% of all foreign-born EU residents [44, 45]—are comparatively underrepresented in the implementation literature. Whether this reflects differences in research infrastructure, publication patterns, or genuinely lower barriers to screening access in high-migration contexts remains an important question for future research. The search was restricted to peer-reviewed literature published in English, which may have led to underrepresentation of implementation experiences documented in other European languages. Furthermore, grey literature was not systematically searched. Reports and implementation documents produced by organizations with direct operational experience of migrant health—including Médecins Sans Frontières, UNHCR, the Refugee Council, and WHO regional offices—represent a substantial evidence base capturing real-world implementation challenges not always reflected in peer-reviewed publications. The role of NGOs and community actors in screening implementation was explicitly addressed in only 3 of the 13 empirical studies, which may reflect an underrepresentation of community-based perspectives in the peer-reviewed literature rather than an absence of NGO involvement in practice—a further argument for the systematic inclusion of grey literature in future reviews. The reliance on qualitative and implementation-focused literature means that quantitative estimates of barrier prevalence cannot be provided. Migrant perspectives, while represented in several studies, may be underrepresented relative to provider viewpoints. Future research should prioritize longitudinal designs tracing migrants’ trajectories through screening pathways, as well as participatory approaches that center migrant voices in intervention co-design.

## Conclusions

In conclusion, addressing gaps in infectious disease screening for migrants requires moving beyond guideline-based approaches toward context-sensitive implementation strategies that integrate policy objectives with health system capacity and migrants’ lived experiences. The evidence synthesized here provides a foundation for developing such strategies and underscores the importance of embedding screening within equitable, accessible, and migrant-centered models of care.

## Additional Files

The additional files for this article can be found as follows:

Supplementary Table 1. Mixed Methods Appraisal Tool (MMAT) quality assessment of the 13 empirical studies included in the thematic synthesis.

Supplementary Table 2. Characteristics of the 36 publications included in the review used to contextualize the findings ordered alphabetically by first author.
