# Supplementary material for: Implementation and Access Gaps in Infectious Disease Screening for Migrants in Europe: A Structured Review with Thematic Synthesis From the ESGITM Group
Source: Ann Glob Health. 2026 Sep 2;92(1):88. doi: 10.5334/aogh.5363 (PMC13544065; doi:10.5334/aogh.5363)
Supplement: Supplementary file 1 [file agh-92-1-5363-s1.pdf]

Supplementary Table 1. Mixed Methods Appraisal Tool (MMAT) quality assessment of the 13 empirical studies included in the thematic synthesis

Study	Country	Study Design	Screening criteria		Design-specific criteria (Q1–Q5)					Score	Overall Quality
			S1	S2	Q1	Q2	Q3	Q4	Q5		
**Nkulu-Kalengayi et al. (2016)**	Sweden	Qualitative	Y	Y	Y	Y	Y	Y	Y	7/7	High
**Louka et al. (2019)**	Greece / Netherlands	Mixed Methods	Y	Y	Y	CT	CT	N	Y	4/7	Moderate
**Kang et al. (2019)**	United Kingdom	Qualitative	Y	Y	Y	Y	Y	Y	Y	7/7	High
**Khanom et al. (2021)**	United Kingdom (Wales)	Qualitative	Y	Y	Y	Y	Y	Y	Y	7/7	High
**Delilovic et al. (2018)**	Sweden	Qualitative	Y	Y	Y	Y	Y	Y	Y	7/7	High
**Gonçalves et al. (2022)**	Spain	Qualitative	Y	Y	Y	Y	Y	Y	Y	7/7	High
**Seedat et al. (2014)**	United Kingdom	Qualitative	Y	Y	Y	Y	Y	Y	Y	7/7	High
**Scott et al. (2022)**	United Kingdom	Quantitative descriptive	Y	Y	CT	N	Y	N	Y	4/7	Moderate
**Carter et al. (2024)**	United Kingdom	Mixed Methods	Y	Y	Y	Y	Y	Y	Y	7/7	High
**Moffat et al. (2023) a**	United Kingdom	Qualitative	Y	Y	CT	CT	Y	Y	CT	4/7	Moderate
**Marrone et al. (2023)**	Italy	Quantitative descriptive	Y	Y	Y	CT	Y	Y	Y	6/7	High
**Kortas et al. (2017) a**	Germany	Quantitative descriptive	Y	Y	Y	CT	Y	CT	Y	5/7	Moderate-High
**Boye et al. (2025) a**	Denmark	Qualitative	Y	Y	Y	Y	Y	Y	CT	6/7	High

Key to ratings:Y = Yes (criterion met), N = No (criterion not met), CT = Cannot tell (insufficient information to judge)

Screening criteria (apply to all study types):S1: Are there clear research questions?, S2: Are the data collection methods adequate to address the research questions?

Design-specific criteria (Q1–Q5):Qualitative (studies 1, 3, 4, 5, 6, 7, 10, 12):Q1 = Relevant recruitment strategy; Q2 = Findings adequately derived from data; Q3 = Interpretation substantiated by data; Q4 = Coherence between data sources, collection, analysis and interpretation; Q5 = Findings transferable

Mixed Methods (studies 2, 9): Q1 = Adequate rationale for mixed methods design; Q2 = Components effectively integrated; Q3 = Integration adequately interpreted; Q4 = Limitations of integration discussed; Q5 = Ethical issues associated with integration discussed

Quantitative Descriptive (studies 8, 11, 13): Q1 = Relevant sampling strategy; Q2 = Sample representative of target population; Q3 = Measurements appropriate; Q4 = Risk of nonresponse bias low; Q5 = Statistical analysis appropriate

Quality thresholds: High = 6–7/7; Moderate-High = 5/7; Moderate = 4/7
