# Supplementary material for: Implementation and Access Gaps in Infectious Disease Screening for Migrants in Europe: A Structured Review with Thematic Synthesis From the ESGITM Group
Source: Ann Glob Health. 2026 Sep 2;92(1):88. doi: 10.5334/aogh.5363 (PMC13544065; doi:10.5334/aogh.5363)
Supplement: Supplementary file 2 [file agh-92-1-5363-s2.pdf]

Supplementary Table 2. Characteristics of the 36 publications included in the review used to contextualise the findings ordered alphabetically by first author.

Notes: Migratory context: 'First arrival' = study conducted in first-arrival/reception settings; 'Long-term settlement' = study conducted in primary care, community or long-term settlement settings; 'Both' = study addresses both stages or is not setting-specific

Author (year)	Country	Setting	Population	Screening focus	Study design	Migratory context
**Aljadeeah et al. (2022)**	Multi-country (Europe)	Across the migratory cycle	Asylum seekers, refugees and undocumented migrants	Access to medicines across the migratory cycle	Scoping review protocol	Both
**Baggaley et al. (2022)**	Europe	Policy/health system level	Migrants broadly	Prevention and treatment of infectious diseases in the universal health coverage era	Policy/conceptual review	Both
**Balaam & Haith-Cooper (2025)**	United Kingdom	Community and refugee support organisations	People seeking asylum and refugees	Vaccine hesitancy — influence of the HARP project	Qualitative study	First arrival
**Bempong et al. (2019)**	Multi-country	Expert meeting	Migrants and refugees broadly	Challenges and solutions for migrant and refugee health	Expert meeting report	Both
**Brandenberger et al. (2019)**	Multi-country (high-income)	Health systems	Migrants and refugees broadly	Healthcare delivery challenges — the 3C model (communication, continuity, confidence)	Systematic literature review	Both
**Carter et al. (2025)**	United Kingdom	Primary care	Migrant patients	Participatory development of migrant health checks (Health Catch-UP! protocol)	Study protocol	Long-term settlement
**Chiarenza et al. (2019)**	Multi-country (Europe)	Health systems under migratory pressure	Refugees and migrants	Supporting healthcare access under high migratory pressure	Policy/implementation report	Both
**Cinardo et al. (2022)**	United Kingdom	Specialist refugee health clinic	Refugees and asylum seekers	Screening for NTDs and other infections	Observational study	First arrival

Author (year)	Country	Setting	Population	Screening focus	Study design	Migratory context
**Coumans & Wark (2024)**	Multi-country	Regional and rural health services	Refugees	Barriers and facilitators to health services in regional/rural areas	Scoping review	Long-term settlement
**Eiset & Wejse (2017)**	Multi-country	Various (reception, primary, secondary care)	Refugees and asylum seekers	Infectious diseases in refugees — current status and future directions	Narrative review	Both
**Griswold et al. (2018)**	Multi-country	Community and health system	Refugee populations broadly	Strengthening preventive services for refugees	Review/conceptual paper	Both
**Hannigan et al. (2016)**	Europe (WHO)	Health system and policy	Migrants broadly	Definitions of 'migrant' and their implications for healthcare access	Policy/conceptual report	Both
**Immordino et al. (2022)**	Multi-country	Health system and policy	Migrants and refugees broadly	COVID-19 vaccination in migrants — lessons learnt and good practices	Review	Both
**Kärki et al. (2014)**	EU/EEA (31 countries)	National health systems	Newly arrived migrants	Infectious disease screening practices across EU/EEA countries	Cross-sectional survey of national practices	First arrival
**Kavukcu & Altintas (2019)**	Multi-country	Refugee settings (reception, camps, clinics)	Healthcare providers in refugee settings	Challenges for healthcare providers in refugee settings	Systematic review	First arrival
**Khan et al. (2016)**	Europe	Policy/political level	Migrants and asylum seekers broadly	Pathogens, prejudice and politics in the European refugee crisis	Commentary/policy paper	First arrival
**Lotfi et al. (2020)**	Multi-country	Health system (guideline level)	Migrants broadly	Quality and reporting of practice guidelines on migrants' health	Methodological review	Both

Author (year)	Country	Setting	Population	Screening focus	Study design	Migratory context
**Matlin et al. (2018)**	Multi-country	Policy/global health level	Migrants and refugees broadly	Migrants' and refugees' health — towards an agenda of solutions	Policy/conceptual paper	Both
**Metersky et al. (2025)**	Multi-country	Across health systems	Migrants broadly	Transnational healthcare practices among migrants — concept analysis	Concept analysis	Both
**Moonen et al. (2023)**	EU/EEA	Various health settings	Migrants broadly	Barriers and facilitators for hepatitis B and C screening	Systematic review	Both
**Noori et al. (2021)**	Europe	Health system and policy level	Migrants and refugees broadly	Strengthening screening and vaccination — closing implementation gaps	Policy/implementation review	Both
**Nowak et al. (2022)**	Europe	Various health and social care settings	Refugees broadly	Healthcare for refugees in Europe — scoping review	Scoping review	Both
**O'Donnell et al. (2008)**	United Kingdom	General practice	Asylum seekers	Asylum seekers' expectations of and trust in general practice	Qualitative study	Long-term settlement
**Pandey et al. (2021)**	Multi-country	Primary and community care	Immigrants with limited English proficiency	Impacts of English language proficiency on healthcare access, use and outcomes	Qualitative study	Both
**Pareek et al. (2019)**	United Kingdom	Primary care	Newly arrived migrants	Community-based multi-disease testing (COMBAT-ID study protocol)	Study protocol	Long-term settlement
**Patel et al. (2021)**	Multi-country	Primary care	Refugees and asylum seekers	Communication experiences	Literature review and narrative synthesis	Both

Author (year)	Country	Setting	Population	Screening focus	Study design	Migratory context
				in primary healthcare		
**Pinheiro et al. (2025)**	Europe	Health system level	Migrants broadly	European guidelines for tuberculosis screening in migrants	Systematic review	Both
**Rast et al. (2025)**	Multi-country	Health and occupational settings	Low-wage migrant workers	Healthcare services for low-wage migrant workers	Systematic review	Long-term settlement
**Robinson et al. (2025)**	Multi-country	Community and health settings	Forced migrant communitie s	Mechanisms in responsive healthcare for forced migrant communities	Qualitative study	Both
**Ross et al. (2019)**	United States	Community organisation s and HIV clinics	Undocumen ted African immigrants	Experiences of HIV testing and linkage to care	Qualitative study	Long-term settlement
**Sagnelli et al. (2018)**	Italy	Outpatient infectious disease clinics, Southern Italy	Undocumen ted immigrants and low-income refugees	HCV chronic infection — clinical findings	Clinical observational study	Long-term settlement
**Santilli (2018)**	France / Italy	Primary care and specialist centres	Sub-Saharan irregular migrants with hepatitis B	Medical care, screening and regularisation of migrants with hepatitis B	Implementation report	Long-term settlement
**Seedat et al. (2018)**	Europe	Various (systematic review)	Migrants broadly	Effectiveness of migrant screening approaches for infectious diseases in Europe	Systematic review	Both
**Suphanchaimat et al. (2015)**	Multi-country	Health systems broadly	Migrants broadly	Challenges in healthcare provision for migrants — providers' perspective	Systematic review	Both
**Tiittala et al. (2018)**	Finland	Reception centres	Asylum seekers	Infectious disease screening implementati on: timing	Implementation/obser vational study	First arrival

Author (year)	Country	Setting	Population	Screening focus	Study design	Migratory context
				and public health response		
**Tosti et al. (2021)**	Italy	Reception centres and primary care	Migrants and asylum seekers upon arrival	Health assessment guidelines upon arrival — Italian national guidelines	Policy/guideline paper	First arrival
